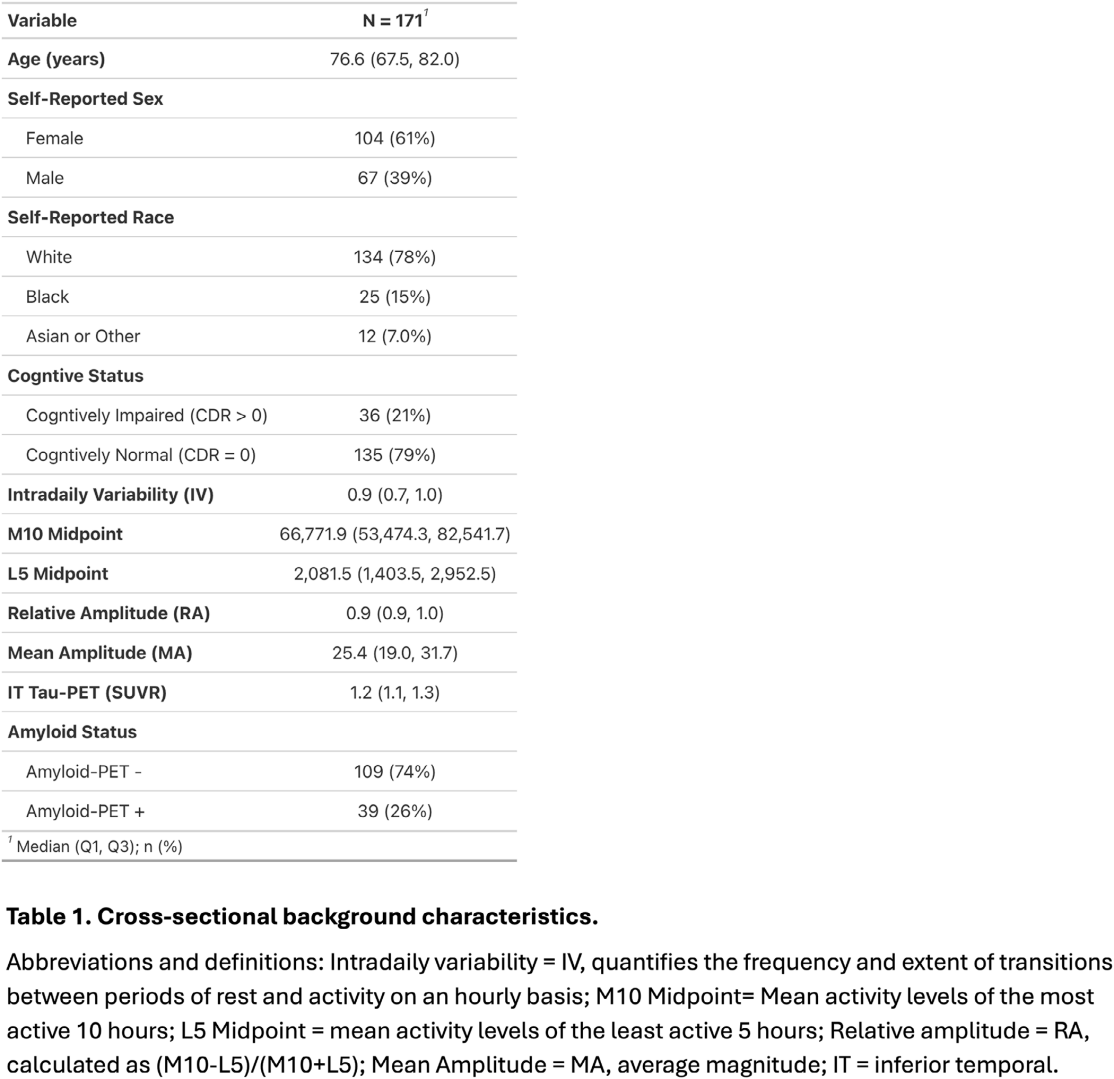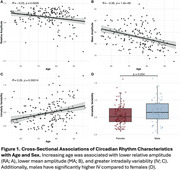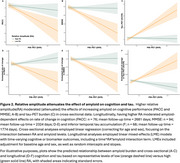# Disruptions in circadian rhythm alter amyloid‐dependent changes in tau and cognition

**DOI:** 10.1002/alz70861_108850

**Published:** 2025-12-23

**Authors:** Yiwen Rao, Valentina Pinilla, Kailee A. Palmgren, Peng Li, Michael J. Properzi, Yazmeen Usman, Victoria Taieb, Eva Pintucci, Era Laho, Gretchen Reynolds, Wai‐Ying Wendy Yau, Ina Djonlagic, Shaun Purcell, Susan Redline, Keith A. Johnson, Reisa A. Sperling, Jasmeer P. Chhatwal, Stephanie A. Schultz

**Affiliations:** ^1^ Department of Neurology, Brigham and Women's Hospital, Boston, MA USA; ^2^ Massachusetts General Hospital, Boston, MA USA; ^3^ Medical Biodynamics Center, Massachusetts General Hospital, Boston, MA USA; ^4^ Mass General Brigham, Boston, MA USA; ^5^ Massachusetts General Hospital, Harvard Medical School, Boston, MA USA; ^6^ Beth Israel Deaconess Hospitals, Harvard Medical School, Boston, MA USA; ^7^ Brigham and Women's Hospital, Harvard Medical School, Boston, MA USA; ^8^ Beth Israel Deaconess Medical Center, Boston, MA USA; ^9^ Harvard T.H. Chan School of Public Health, Boston, MA USA; ^10^ Brigham and Women’s Hospital, Harvard Medical School, Boston, MA USA

## Abstract

**Background:**

The circadian system drives biological and physiological processes aligning with a near 24‐h light‐dark cycle, referred to as circadian rhythms (CR). Disruptions in CR are an emerging feature of aging and Alzheimer disease (AD). Alterations in CR ‐ including increased fragmentation of rest‐activity rhythm (RAR) and decreased amplitude of RAR ‐ are hypothesized to be contributors to AD pathogenesis and potentially modifiable risk factors for AD. Here we examined whether RAR features might be associated with amyloid‐dependent cognitive and tau changes in a cohort of cognitively normal (CN) and cognitively impaired (CI) older adults.

**Methods:**

CR analysis was performed on actigraphy data using nonparametric analyses and a data adaptive approach (empirical mode decomposition) to measure RAR fragmentation (intradaily variability [IV]), relative amplitude (RA), and mean amplitude (MA) in 171 individuals (*n* =135 CN and n=36 CI; Table 1). Individuals completed cross‐sectional and longitudinal (in a subset of n=76) cognitive assessments (PACC and MMSE), tau‐positron emission tomography (PET), and amyloid‐PET.

**Results:**

Cross‐sectionally, increasing age was associated with lower RA (r=‐0.23, *p* =0.0029; Figure 1A) and MA (r=‐0.36, *p* =1.2e‐06; Figure 1B), and higher IV (r=0.29, *p* =0.0001; Figure 1C), and male sex was associated with higher IV (*p* =0.034; Figure 1D). Using linear regressions, adjusting for age and sex, higher RA attenuated the effect of amyloid on and PACC scores (RA*amyloid: B[SE]=24.55[12.1], *p* =0.044; Figure 2A), MMSE scores (RA*amyloid: B[SE]=76.12[24.4], *p* =2.15e‐03; Figure 2B) and inferior temporal tau‐PET levels (RA*amyloid: B[SE]=‐13.36[2.7], *p* =2.43e‐06; Figure 2C). Using linear mixed effect models, adjusting for baseline age and sex, higher RA modified the effect of baseline amyloid on longitudinal PACC decline (time*RA*amyloid: B[SE]=3.16[1.1], *p* =0.007; Figure 2D), MMSE decline (time*RA*amyloid: B[SE]= 9.56[3.2], *p* =0.036; Figure 2E), and tau accumulation (time*RA*amyloid: B[SE]=‐0.56[0.2], *p* =0.016; Figure 2D).

**Conclusion:**

In a sample composed of CN and CI individuals, disruptions in CR measures were associated with older age and male sex. Higher relative amplitude of a RAR was associated with diminished cross‐sectional and longitudinal tau accumulation and cognitive decline in people with elevated amyloid. These findings suggest robust CR may provide resilience to amyloid‐related cognitive decline and tau accumulation and supports circadian dysfunction as a potentially modifiable risk factor for AD progression.